# The mediator role of resilience between psychological predictors and health-related quality of life in breast cancer survivors: a cross-sectional study

**DOI:** 10.1186/s12885-022-09177-0

**Published:** 2022-01-12

**Authors:** Kaina Zhou, Fan Ning, Wen Wang, Xiaomei Li

**Affiliations:** School of Nursing, Xi’an Jiaotong University Health Science Centre, No. 76 Yanta West Road, Xi’an, 710061 Shaanxi China

**Keywords:** Breast cancer, Coping style, Health-related quality of life, Perceived social support, Resilience

## Abstract

**Background:**

Although many psychological factors have been associated with health-related quality of life (HRQoL), the mediator role of resilience between psychological predictors (i.e., coping styles and perceived social support) and HRQoL has rarely been explored in breast cancer survivors (BCSs).

**Methods:**

A total of 231 BCSs participated in this cross-sectional survey. Pearson correlation was performed to analyze the relationships among coping styles (confrontation, avoidance, and resignation), perceived social support, resilience, and HRQoL. A multivariate linear regression analysis was applied to identify the psychological predictors of HRQoL and resilience, respectively. A structural equation modeling (SEM) was employed to examine the mediating role of resilience between coping styles, perceived social support, and HRQoL.

**Results:**

Perceived social support and resilience were positively associated with confrontation. Resilience was positively associated with perceived social support. HRQoL had positive correlations with confrontation/avoidance, perceived social support, and resilience. Resilience and resignation/avoidance were significant independent predictors of HRQoL, while resignation/confrontation and perceived social support were significant independent predictors of resilience. Confrontation/resignation, perceived social support, and resilience had significant direct effects on HRQoL; confrontation/resignation and perceived social support had significant direct effects on resilience; resilience had significant mediator roles between confrontation/resignation, perceived social support, and HRQoL.

**Conclusions:**

Resilience was a significant mediator between coping styles, perceived social support, and HRQoL. A resilience-oriented intervention is recommended to alleviate the detrimental influences of low resilience on HRQoL, providing a new strategy for improving the health status of BCSs.

## Background

Breast cancer is the most common malignant tumor in women worldwide [1, 2]. Earlier diagnosis via screening and advances in treatment strategies have led to improvements by 5-year and 10-year overall survival (ranging from 80 to 90%) [3]. However, a large number of breast cancer survivors (BCSs) continue to suffer from long-term psychosomatic trauma, caused by the disease itself and by treatment-related adverse effects, which lead to reduced levels of health-related quality of life (HRQoL) [4–7].

Many studies have reported the risk factors of HRQoL, which include socio-demographics, clinical characteristics, treatment-related/surgical factors, and behavioral and psychosocial factors [8, 9]. With the increased survival rate, health professionals have paid more attention to the psychological rehabilitation of BCSs [4]. The variables most commonly focused on were social support, psychological symptoms, emotional functioning, coping styles, confidence and self-efficacy, and future perspective and appraisal [8, 10].

Apart from the above-mentioned variables reported by systematic reviews, resilience and perceived social support are also factors related to HRQoL [11, 12], along with higher resilience and perceived social support were associated with better HRQoL [11, 13]. Resilience is regarded as the most important factor that should be assessed at the time of breast cancer diagnosis, which allows early identification of patients who may need more psychological support [11].

Although many psychological factors are related to HRQoL, the correlation among these factors and their role mechanisms to HRQoL have rarely been explored [8–13]. Considering the importance of resilience at the early stage of breast cancer rehabilitation [11], it is imperative to identify the psychological predictors of resilience, especially the variables reflecting personal coping styles and perception of social support. On this basis, the mediator role of resilience between these psychological factors and HRQoL will be further clarified.

The purpose of this study was to identify the mediator role of resilience between coping styles, perceived social support, and HRQoL. The three hypotheses proposed in this study are: (1) coping styles, perceived social support, and resilience are independent predictors of HRQoL; (2) coping styles and perceived social support are independent predictors of resilience; and (3) resilience mediates the relationship between coping styles, perceived social support, and HRQoL. The study’s findings will provide new evidence for identifying the mediator role of resilience in the relationship between psychological factors and HRQoL, and for developing resilience-oriented intervention programs, taking into account positive coping and social support perception to improve HRQoL in BCSs.

## Methods

### Design

This was a cross-sectional study.

### Participants and sampling

Convenience sampling was applied to recruit female BCSs who were attending a follow-up program at two general hospitals in Xi’an. Inclusion criteria: Chinese speakers of age 18 years or older, who had had surgical treatment and adjuvant therapy (e.g., chemotherapy, radiotherapy) for breast cancer for more than 1 month, prior to participating in the study. Exclusion criteria: women with cognitive disorders (screened by a blinded psychiatrist according to the DSM-5 criteria), comorbid non-breast tumors, or other types of breast disease.

### Sample size

Based on the metric of 5 to 10 subjects per item in a validated instrument to ensure sufficient power [14], and considering that the largest instrument used had 36 items, the appropriate sample size of the study was estimated to be between 180 to 360 participants.

### Measurements

#### Medical Coping Mode Questionnaire

The 20-item Chinese Medical Coping Mode Questionnaire (MCMQ) has three subscales, namely, confrontation, avoidance, and resignation. This scale has been validated in patients with chronic disease and has satisfactory psychometric properties [15]. In this study, the Cronbach’s α was 0.72 (confrontation), 0.71 (avoidance), and 0.90 (resignation).

#### Multidimensional Scale of Perceived Social Support

The 12-item Chinese Multidimensional Scale of Perceived Social Support (MSPSS) has a total score ranging from 12 to 84, with a higher score representing higher perceived social support [16]. The Chinese MSPSS has been validated in patients with methadone maintenance treatment (Cronbach’s α = 0.92) [17] and in university students (Cronbach’s α = 0.92) [18]. In this study, the Cronbach’s α was 0.90.

#### Connor-Davidson Resilience Scale

The total score of the 25-item Chinese Connor-Davidson Resilience Scale (CD-RISC) ranges from 0 to 100, with a higher score indicating higher resilience. The Chinese CD-RISC has been validated with satisfactory psychometrics [19]. In this study, the Cronbach’s α was 0.96.

#### Functional Assessment of Cancer Therapy-Breast version 4.0

The 36-item Chinese Functional Assessment of Cancer Therapy-Breast version 4.0 (FACT-Bv4.0) has five subscales, namely physical well-being, functional well-being, social/family well-being, emotional well-being, and breast-cancer-specific concerns [20], with a higher total score (ranging from 0 to 144) reflecting better HRQoL [20]. The Chinese FACT-Bv4.0 has been well validated in breast cancer patients [20]. In this study, the Cronbach’s α was 0.95.

### Data collection

Data were collected from September 2020 to June 2021. The BCSs were instructed to complete the questionnaires independently. If the BCSs had difficulties in writing or reading, a trained data collector read the items to them and recorded their responses.

### Data analysis

Continuous variables were summarized by mean (*M*) and standard deviation (*SD*), while categorical variables were summarized by frequencies and percentages. The normality of the scores regarding resilience, perceived social support, coping style (i.e., confrontation, avoidance, and resignation), and HRQoL were tested using the Shapiro-Wilk method. Pearson correlation was performed to analyze the relationships among coping styles, perceived social support, resilience, and HRQoL. A multivariate linear regression analysis was applied to identify the predictors of HRQoL and resilience, respectively. Structural equation modeling (SEM) was employed using the maximum likelihood bootstrapping method to examine the mediating role of resilience between coping styles, perceived social support, and HRQoL. Standard direct, indirect, total effects, and R^2^ with a corresponding 95% bias-corrected confidence interval (CI) were estimated based on 1000 random samples (bootstrapping random sample) generated by a computer [21, 22]. The data were analyzed using SPSS 25.0 and AMOS 26.0 (IBM Corp., Armonk, NY). A value of *p* < .05 (two-tailed) was considered statistically significant.

### Ethical statement

The study protocol (2020–1145) was reviewed and approved by the Human Research Ethics Committee of Xi’an Jiaotong University. Before administering the survey questionnaires, written informed consent was obtained from each participant. Additionally, the study conforms to the standards held by the Declaration of Helsinki.

## Results

**Fig. 1:**
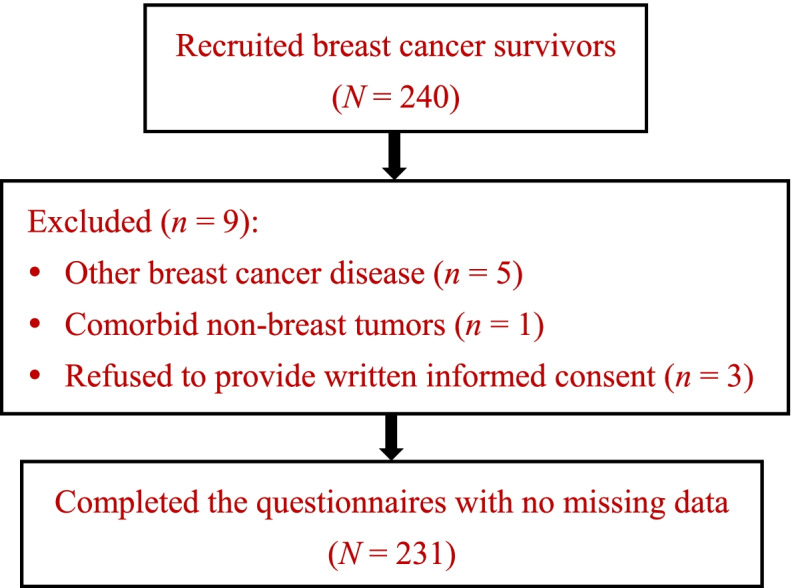
Flow diagram

Two hundred forty eligible BCSs were recruited in the study, and 231 (96.3%) completed the questionnaires with no missing data. Nine BCSs were excluded, due to having had other types of breast cancer disease (*n* = 5), comorbid non-breast tumors (*n* = 1), and refusal to provide written informed consent (*n* = 3) (Fig. 1). The BCSs’ socio-demographics, clinical characteristics, and the scores of coping styles (i.e., confrontation, avoidance and resignation), perceived social support, resilience and HRQoL are shown in Table 1.

**Table 1:** Socio-demographics, clinical characteristics, psychological factors, and health-related quality of life (HRQoL) (*N* = 231)

	*n* (%)
**Socio-demographics**	
Age (years) (*M*) (*SD*); range: 25–78	(48.15) (10.28)
Education level	
Primary and below	48 (20.8)
Secondary	149 (64.5)
Tertiary	34 (14.7)
Marital status	
Married	228 (98.7)
Other marital status	3 (1.3)
Has children	
Yes	223 (96.5)
No	8 (3.5)
Employment status	
Unemployed	91 (39.4)
Retired	29 (12.6)
Employed	111 (48.1)
Average monthly income (Chinese Yuan)	
< 3000	91 (39.4)
3000–6000	104 (45.0)
6001–9000	28 (12.1)
> 9000	8 (3.5)
Residence place	
Urban	111 (48.1)
Rural	120 (51.9)
Chronic disease (disease course > 6 months)	
Yes	15 (6.5)
No	216 (93.5)
**Clinical characteristics**	
Illness stage	
I	72 (31.2)
II	109 (47.2)
III	35 (15.2)
IV	15 (6.5)
Metastasis	
Yes	78 (33.8)
No	153 (66.2)
Surgery	
Yes	199 (86.1)
No	32 (13.9)
Neoadjuvant chemotherapy	
Yes	14 (6.1)
No	217 (93.9)
Adjuvant therapy	
Chemotherapy	
Yes	204 (88.3)
No	27 (11.7)
Radiotherapy	
Yes	81 (35.1)
No	150 (64.9)
Endocrine therapy	
Yes	124 (53.7)
No	107 (46.3)
Targeted therapy	
Yes	25 (10.8)
No	206 (89.2)
**Psychological factors (*M*) (*SD*)**	
Resilience	(52.60) (12.15)
Perceived social support	(66.50) (9.28)
Coping styles	
Confrontation	(19.05) (3.61)
Avoidance	(16.96) (1.78)
Resignation	(10.82) (2.59)
**HRQoL (*M*) (*SD*)**	(85.62) (17.87)

*M* Mean, *SD* Standard deviation

The normality test showed that the scores for resilience (*W* = 0.99, *p* = .23), perceived social support (*W* = 0.98, *p* = .21), confrontation (*W* = 0.98, *p* = .21), avoidance (*W* = 0.99, *p* = .23), resignation (*W* = 0.98, *p* = .21) and HRQoL (*W* = 0.99, *p* = .25) corresponded to the normal distribution.

Except for avoidance, perceived social support, and resilience were positively associated with confrontation (*r* = 0.35, *p* < .01; *r* = 0.53, *p* < .01) and negatively associated with resignation (*r* = − 0.49, *p* < .01; *r* = − 0.66, *p* < .01), respectively. Resilience was positively associated with perceived social support (*r* = 0.59, *p* < .01). HRQoL had positive correlations with confrontation (*r* = 0.48, *p* < .01)/avoidance (*r* = 0.17, *p* < .05), perceived social support (*r* = 0.52, *p* < .01), and resilience (*r* = 0.72, *p* < .01), but had negative correlations with resignation (*r* = − 0.67, *p* < .01) (Table 2).

**Table 2:** Correlations among coping styles, perceived social support, resilience, and health-related quality of life (HRQoL) (*N* = 231)

	Confrontation	Avoidance	Resignation	Perceived social support	Resilience
**Coping styles**					
Confrontation	1				
Avoidance	0.18^**^	1			
Resignation	−0.58^**^	0.07	1		
**Perceived social support**	0.35^**^	0.11	−0.49^**^	1	
**Resilience**	0.53^**^	0.12	−0.66^**^	0.59^**^	1
**HRQoL**	0.48^**^	0.17^*^	−0.67^**^	0.52^**^	0.72^**^

^*^p < .05. ^**^p < .01

Multivariate stepwise linear regression analysis further indicated that resilience [*B* = 0.66, 95% CI (0.51, 0.80);, *p* < .001] and resignation [*B =* − 2.08 95% CI (− 2.82, − 1.35); *p* < .001]/avoidance [*B* = 1.15, 95% CI (0.34, 1.95), *p* = .005] were significant independent predictors of HRQoL; while resignation [*B =* -2.00, 95% CI (− 2.54, − 1.46), *p* < .001]/confrontation [*B =* 0.83, 95% CI (0.43, 1.23), *p* < .001] and perceived social support [*B =* 0.46, 95% CI (0.31, 0.61), *p* < .001] were significant independent predictors of resilience (Table 3).

**Table 3:** Predictors of health-related quality of life (HRQoL) and resilience: a multivariate stepwise linear regression analysis (*N* = 231)

Dependents	Predictors	*B* (95% CI)	*P*	VIF
HRQoL	Resilience	0.66 (0.51, 0.80)	< .001	2.03
	Resignation	−2.08 (− 2.82, −1.35)	< .001	2.01
	Residence place (ref. urban)	6.19 (3.04, 9.33)	< .001	1.12
	Avoidance	1.15 (0.34, 1.95)	0005	1.07
	Illness stage (ref. I)	−2.60 (−4.47, −0.73)	.007	1.15
	*R* = 0.80, *R*^2^ = 0.63, *R*^2^_adj_ = 0.63, *F* = 77.90, *p* < .001			
Resilience	Resignation	−2.00 (− 2.54, − 1.46)	<.001	1.73
	Perceived social support	0.46 (0.31, 0.61)	<.001	1.35
	Endocrine therapy (ref. yes)	−6.19 (−8.63, −3.73)	<.001	1.07
	Confrontation	0.83 (0.43, 1.23)	<.001	1.60
	Residence place (ref. urban)	−3.67 (−6.15, −1.19)	.004	1.10
	Chronic disease (ref. yes)	−5.71 (− 10.78, −0.64)	.027	1.12
	*R* = 0.79, *R*^2^ = 0.63, *R*^2^_adj_ = 0.62, *F* = 62.37, *p* < .001			

The independents were variables shown in Table 1: education level (ref. primary and below), marital status (ref. married), has children (ref. yes), employment status (ref. unemployed), average monthly income (Chinese Yuan) (ref. < 3000), residence place (ref. urban), chronic disease (ref. yes), illness stage (ref. I), metastasis (ref. yes), surgery (ref. yes), chemotherapy (ref. yes), neoadjuvant chemotherapy (ref. yes), radiotherapy (ref. yes), endocrine therapy (ref. yes), targeted therapy (ref. yes), and continuous characteristics (age, resilience, perceived social support, confrontation, avoidance, resignation)

*R*^*2*^_*adj*_ Adjusted *R*^2^, *95%CI* 95% confidence interval, *VIF* Variance inflation factor (reference value: < 10)

The three models of the SEM analysis showed that confrontation/resignation, perceived social support, and resilience had significant direct effects on HRQoL; confrontation/resignation and perceived social support had significant direct effects on resilience; and resilience had a significant mediator role between confrontation/resignation, perceived social support, and HRQoL (Table 4).

**Table 4:** Standardized mediator effect of resilience, and standardized direct/indirect/total effect of coping styles and perceived social support on health-related quality of life (*N* = 231)

Standardized effect (95% CI)	Mediator (resilience)	*P*	HRQoL	*P*
**Model A**				
Coping styles (confrontation)				
Direct	0.37 (0.26, 0.48)	.001	0.14 (0.05, 0.22)	.006
Indirect	–	–	0.21 (0.15, 0.27)	.001
Total	0.37 (0.26, 0.48)	.001	0.35 (0.24, 0.44)	.002
Perceived social support				
Direct	0.46 (0.34, 0.57)	.003	0.13 (0.03, 0.22)	.011
Indirect	–	–	0.26 (0.19, 0.35)	.002
Total	0.46 (0.34, 0.57)	.003	0.40 (0.27, 0.50)	.003
Resilience				
Direct	–	–	0.57 (0.48, 0.66)	.002
Indirect	–	–	–	–
Total	–	–	0.57 (0.48, 0.66)	.002
*R*^2^ (95% CI)	0.47 (0.34, 0.55)	.006	0.54 (0.44, 0.62)	.004
**Model B**				
Coping styles (avoidance)				
Direct	0.05 (−0.04, 0.15)	.248	0.08 (− 0.02, 0.17)	.160
Indirect	–	–	0.03 (−0.03, 0.10)	.246
Total	0.05 (−0.04, 0.15)	.248	0.11 (0.004, 0.22)	.040
Perceived social support				
Direct	0.59 (0.47, 0.67)	.004	0.14 (0.03, 0.22)	.007
Indirect	–	–	0.37 (0.29, 0.45)	.002
Total	0.59 (0.47, 0.67)	.004	0.50 (0.39, 0.59)	.004
Resilience				
Direct	–	–	0.63 (0.54, 0.71)	.002
Indirect	–	–	–	–
Total	–	–	0.63 (0.54, 0.71)	.002
*R*^2^ (95% CI)	0.35 (0.22, 0.45)	.006	0.53 (0.43, 0.62)	.005
**Model C**				
Coping styles (resignation)				
Direct	−0.49 (− 0.58, − 0.40)	.002	− 0.33 (− 0.43, − 0.23)	.002
Indirect	–	–	− 0.22 (− 0.28, − 0.16)	.001
Total	− 0.49 (− 0.58, − 0.40)	.002	−0.55 (− 0.64, − 0.45)	.002
Perceived social support				
Direct	0.36 (0.24, 0.46)	.003	0.09 (0.002, 0.18)	.047
Indirect	–	–	0.16 (0.10, 0.22)	.002
Total	0.36 (0.24, 0.46)	.003	0.25 (0.14, 0.35)	.003
Resilience				
Direct	–	–	0.44 (0.34, 0.54)	.002
Indirect	–	–	–	–
Total	–	–	0.44 (0.34, 0.54)	.002
*R*^2^ (95% CI)	0.53 (0.41, 0.61)	.005	0.59 (0.50, 0.66)	.004

*95% CI* 95% confidence interval

## Discussion

The findings of this study indicated that coping styles, perceived social support, and resilience were predictors of HRQoL; resilience was influenced by coping styles and perceived social support, and had a mediating role between coping styles, perceived social support, and HRQoL.

The correlation findings demonstrated that confrontation was associated with better perceived social support and resilience, and better perceived social support was associated with higher resilience. This reflects the fact that positive psychological states can be shown in different aspects, and are positively correlated with each other [23]. Consistent with previous reports, it was found that confrontation, perceived social support, and resilience were positively associated with HRQoL, which indicated that a sound psychological state was beneficial to HRQoL improvement [8–12]. Although avoidance is a negative coping style, it was still helpful in protecting the BCSs’ psychological defense system, which allowed more time for them to recover from their distress [24].

The multivariate linear regression analysis found that perceived social support was not a significant predictor of HRQoL when sociodemographic factors, clinical characteristics, and other psychological factors were considered. This is inconsistent with previous reports [12, 13]. Although we found that perceived social support was significantly positively correlated with HRQoL, it could be influenced by negative psychological factors, such as resignation. After we excluded the resignation variable from the regression model, perceived social support was found to be a significant predictor of HRQoL. This finding indicates that it is important to pay attention to negative psychological states, which will help in avoiding the weakening or offsetting effects on the benefits of positive psychological states.

Confrontation and perceived social support were positive predictors of resilience, while resignation was a negative predictor. This suggests that interventions should focus on coping styles and perception of social support while managing resilience, as suggested by the findings of similar studies [12, 13, 25]. Resilience was found to be lower in BCSs who were not receiving endocrine therapy, were living in a rural location, and had no chronic disease. This is probably because: (1) the BCSs not receiving endocrine therapy might have a special pathological type that is not suitable for receiving endocrine therapy, which would lead them to worry about their prognosis, and consequently decrease their resilience; (2) the BCSs living in rural locations might lack information about treatment, prognosis, and self-care, which would lead to a sense of uncertainty and lower their resilience; (3) the BCSs with no chronic disease who had good health status, may experience severe psychological distress after being diagnosed with breast cancer, which would lead to low resilience. Although the above characteristics of BCSs had no clear relationship with low resilience on the surface, their poor psychological states might be related closely to low resilience [26–28]. Thus, healthcare providers should pay more attention to BCSs with these characteristics, to enhance their resilience and support the improvement of their HRQoL.

Resilience was a significant mediator between confrontation/resignation, perceived social support, and HRQoL, indicating that it has an important role in strengthening the positive influences of confrontation and perceived social support, or weakening the negative influences of resignation, on HRQoL. The findings of this study further support the role of resilience, that is, an individual’s ability to adapt and successfully cope with adversity [11, 29–32]. The final SEM results support the three hypotheses of this study and suggest that strengthening resilience would enhance the intervention effects regarding coping styles and perception of social support, which will improve HRQoL.

Although avoidance was positively correlated with HRQoL, it had no significant direct effect on resilience and no significant indirect effect on HRQoL via resilience. This is probably due to the weak correlation between these variables in our study sample. Considering the protecting role of avoidance under certain circumstances, the influences of avoidance on resilience and HRQoL should be further explored.

The study findings have important implications for clinical practice and the development of intervention programs to improve resilience and the HRQoL of BCSs. According to our findings, more resources regarding psychological support should be provided in health management programs for BCSs. Additionally, healthcare providers should focus more on resilience and coping while informing the patient of the breast cancer diagnosis, which would help decrease the patient’s psychological trauma during the treatment and long-term rehabilitation process from the beginning. Moreover, the findings also suggest that resilience-oriented interventions would be effective in alleviating the detrimental influences of low resilience on HRQoL, providing a new strategy for improving health status regarding BCSs.

The study had some limitations. First, since coping styles, perceived social support, resilience and HRQoL were measured using self-reported data, the relationships among these variables might be susceptible to response bias. Second, causal relationships could not be identified due to the cross-sectional design. Longitudinal studies are recommended in future work to further explore the relationship trajectories during long-term rehabilitation. Third, the study was conducted in Xi’an, which limited the generalizability of the findings.

## Conclusions

Our findings indicate that coping styles, perceived social support, and resilience were predictors of HRQoL, and that resilience was a significant mediator between coping styles, perceived social support, and HRQoL. Resilience-oriented intervention programs should be developed that focus on improving the positive effect of coping styles and perceived social support on the HRQoL of BCSs.

## Data Availability

The data that support the findings of this study are available from the corresponding author upon reasonable request. The data are not publicly available due to privacy or ethical restrictions.

## Abbreviations

HRQoL: Health-related quality of life
BCSs: Breast cancer survivors
SEM: Structural equation modeling
CI: Confidence interval
MCMQ: Medical Coping Mode Questionnaire
MSPSS: Multidimensional Scale of Perceived Social Support
CD-RISC: Connor-Davidson Resilience Scale
FACT-Bv4.0: Functional Assessment of Cancer Therapy-Breast version 4.0
SD: Standard deviation
